# Rare Presentation of Unilateral Weakness, Involuntary Movements and Ataxia with Subcortical T2 Hypointensity in a Diabetic Patient: A Case Report

**DOI:** 10.1155/2012/768189

**Published:** 2012-09-23

**Authors:** Venkatraman Indiran, Prabakaran Maduraimuthu

**Affiliations:** Department of Radiodiagnosis, Sree Balaji Medical College & Hospital, 7 Works Road, Chromepet, Tamil Nadu, Chennai 600044, India

## Abstract

Subcortical T2 hypointensity on MRI is not a common finding. We present a case of subcortical T2 hypointensity in a diabetic patient, who was referred with weakness of left lower limb and involuntary movements and ataxia of the left upper limb. Lab reports confirmed the diagnosis of nonketotic hyperglycemia. It is rather important to identify subcortical T2 hypointensity which has only been recently found to be associated with nonketotic hyperglycemia. Early identification and prompt correction of blood sugar would help in alleviating the neurological symptoms.

## 1. Introduction

There are numerous causes for subcortical T2 hyperintensity on MR images, which is quite a common finding. However, subcortical white matter T2 hypointensity is an uncommon finding which has been described as an uncommon manifestation of ischemia, multiple sclerosis, and meningitis. There have been recent reports of subcortical white matter T2 hypointensity being associated with nonketotic hyperglycemia. Nonketotic hyperglycemia (NKH) is a clinical syndrome characterized by severe hyperglycemia and hyperosmolarity, with absence of ketoacidosis. Nonketotic hyperglycemic patients present with neurological abnormalities including hemi chorea, seizure, hemianopsia, and coma [1, 2]. Our patient presented with weakness of left lower limb, involuntary movements, and ataxia of the left upper limb.

## 2. Case Report

**Figure 1:**
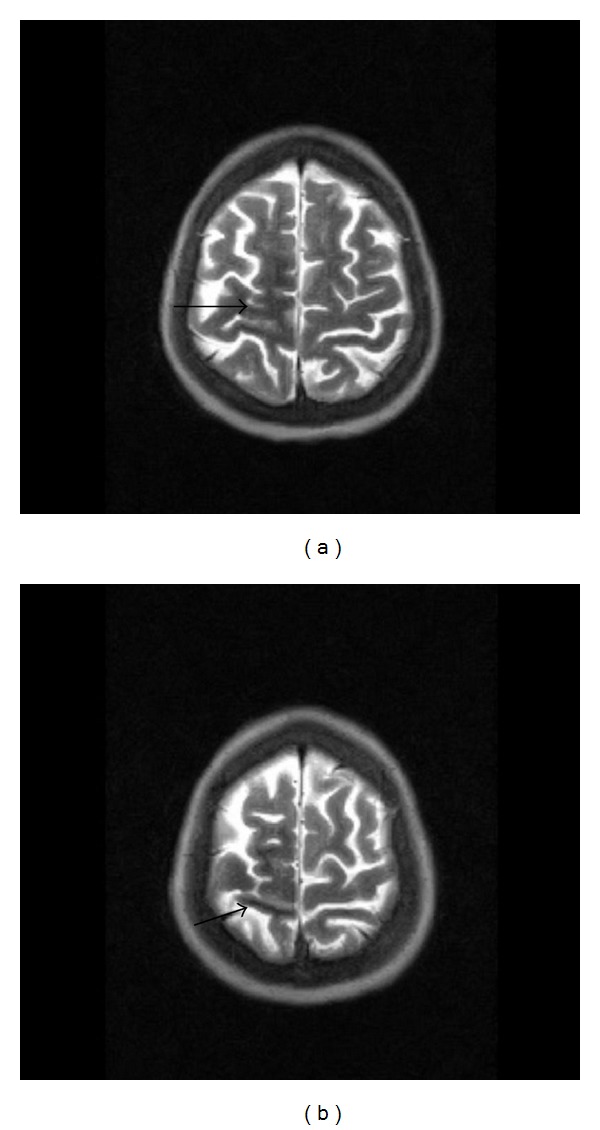
Axial T2 image shows subcortical T2 hypointensity in right pre- and postcentral white matter.

**Figure 2:**
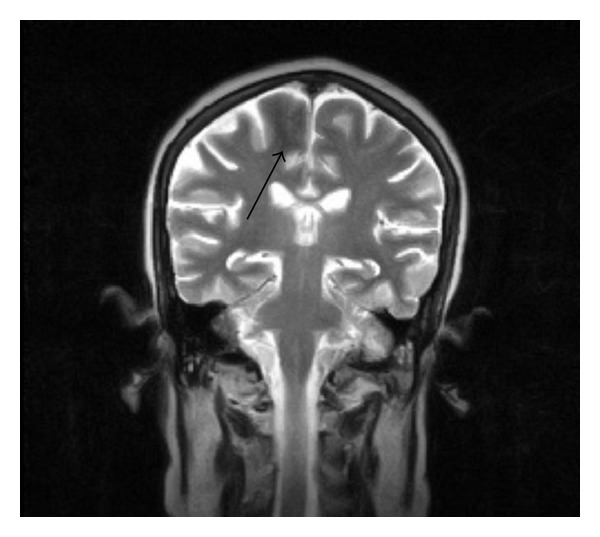
Coronal T2 image shows subcortical T2 hypointensity in right postcentral white matter.

**Figure 3:**
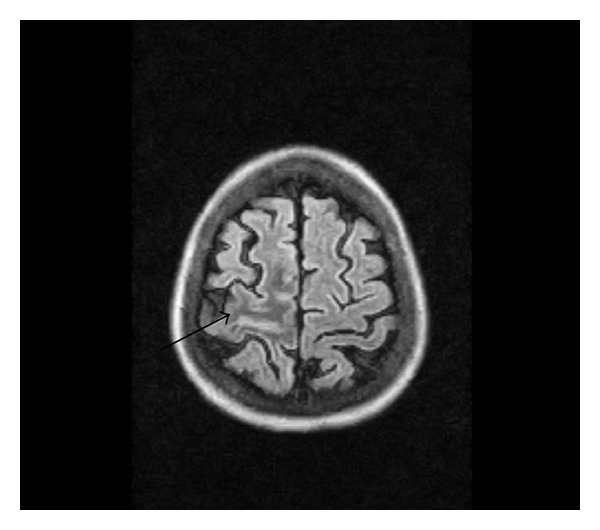
Axial T2 FLAIR image shows subcortical T2 hypointensity in right pre- and postcentral white matter.

**Figure 4:**
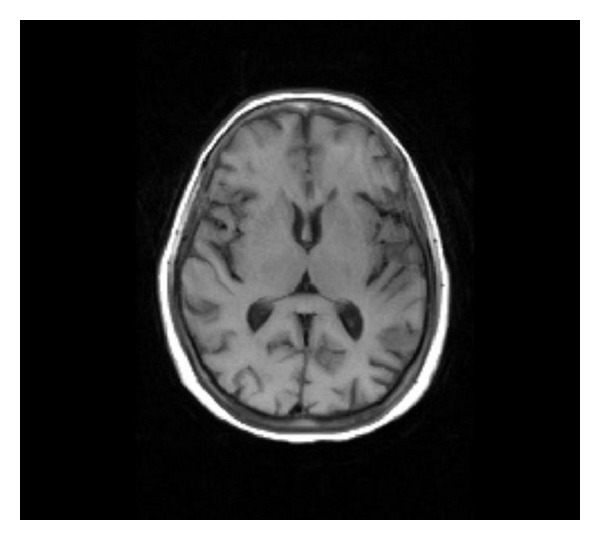
Axial T1 image shows normal basal ganglia.

**Figure 5:**
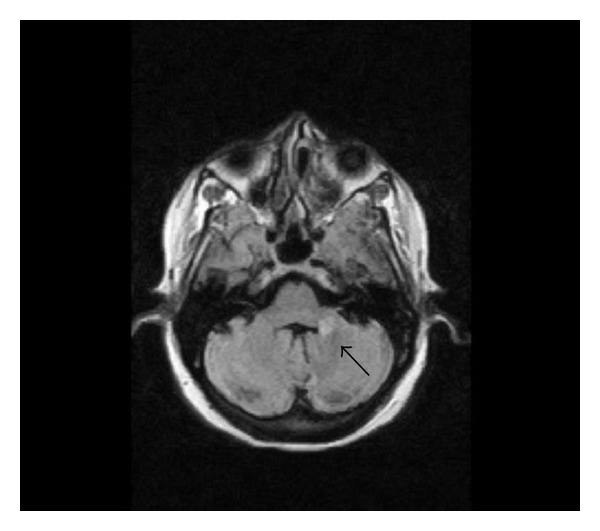
Axial T2 FLAIR image shows small hyperintense lesion in the left middle cerebellar peduncle.

52-year-old female patient with poorly controlled type II diabetes mellitus was referred as outpatient for an MRI brain study with complaints of acute onset of weakness of left lower limb with involuntary movements (hemi chorea hemiballismus) and ataxia of the left upper limb, for a period of 4 days. There was no history of seizures. Patient did not have fever, neck stiffness, or photophobia. There was no similar past history. There was no history of any known malignancy. There was no relevant family history. Patient's laboratory reports revealed very high blood sugar levels (fasting blood sugar 578 mg/dL and postprandial blood sugar 678 mg/dL) with absence of urine ketones. Renal parameters were abnormal (blood urea 108 mg/dL and serum creatinine 1.9 mg/dL). MRI brain study (0.4 Tesla Aperto; Hitachi Medical Systems Inc., Ohio, US) revealed subcortical T2 hypointensity in the right pre- and postcentral subcortical white matter on T2 and T2 FLAIR images with minimal sulcal hyperintensity in the right central sulcus (Figures 1(a), 1(b), 2, and 3). Diffusion-weighted (DW) images and apparent diffusion coefficient (ADC) maps showed no restricted diffusion. No obvious abnormality/signal change was visualized in the basal ganglia region on T1-weighted images (Figure 4). A T2 FLAIR hyperintense lesion measuring ~1 cm is seen in the left middle cerebellar peduncle (Figure 5). Intravenous gadolinium was not given as the renal parameters were not within normal limits. The patient had reasonable improvement of symptoms after correction of hyperglycemia, when interviewed after 2 weeks.

## 3. Discussion

Nonketotic hyperglycemia is usually observed in patients over age 50 with type 2 diabetes mellitus (type 2 DM).

Nonketotic hyperglycemia has been associated with various neurological manifestations ranging from delirium, partial or generalized seizures, hemichorea-hemiballism, dysphagia, hemianopsia, hemiparesis, and hemisensory loss [1, 2]. Hemiballism-hemichorea caused by nonketotic hyperglycemia was first reported by Bedwell in 1960. He described a 65-year-old woman who developed ballistic movements in all four limbs during three episodes of hyperglycemia [3].

Possible hypotheses are depletion of the inhibitory neurotransmitter gamma-aminobutyric acid (GABA), which is metabolized in the brain as an energy source in NKH with deficiency of GABA in the basal ganglia leading to HC-HB, while in the cerebral cortex it may lower the seizure threshold.

Neuroimaging findings in NKH-related neurological disorders are varied. In those with HC-HB, the imaging abnormalities include increased densities on CT and hyperintense lesions on T1-weighted MRI in the contralateral basal ganglia [4, 5].

The imaging findings are usually at the contralateral basal ganglia in cases of unilateral presentation. However, in our case basal ganglia were normal.

Another hypothesis is hyperglycemia inducing transient focal cerebral ischemia [6]. Subcortical T2 hypointensity as a feature of early cortical ischemia has been identified in animal studies [7]. Various causes of subcortical T2 hypointensity are viral encephalitis, meningitis, leptomeningeal metastasis, hemorrhagic infarct, and hypoxic insult in acute setting and multiple sclerosis, Sturge Weber syndrome, Moyamoya disease, tuberous sclerosis, and hemimegalencephaly in the chronic setting as presented in the table by Raghavendra et al. [8].

Ataxia in our case could have been due to lacunar infarct represented by T2 FLAIR hyperintense lesion in left middle cerebellar peduncle.

No appropriate cause could be identified for the HB-HC in our case. Based on the recent reports available, hemichorea-hemiballismus associated with putaminal abnormality on CT and MR appears to be the common presentation in cases of NKH.

Incidence of subcortical T2 hypointensity with associated seizures in nonketotic hyperglycemia has been reported [8]. However subcortical T2 hypointensity with associated hemi paresis has not yet been described to the best of our knowledge.

Recognition of the association of these neurological and radiological abnormalities with NKH is important because correction of the underlying hyperglycemia will lead to rapid improvement.
